# A Case Report of Three Patients Who Underwent Temporary Peripheral Nerve Stimulation for Treatment of Knee Pain Secondary to Osteoarthritis

**DOI:** 10.7759/cureus.40473

**Published:** 2023-06-15

**Authors:** Cheng-Cheng Zhu, Akshat Gargya, Naeem Haider

**Affiliations:** 1 Anesthesiology and Pain Management, The Robert Larner, M.D. College of Medicine, University of Vermont, Burlington, USA

**Keywords:** neuromodulation, knee pain, case series, ultrasound, peripheral nerve stimulation, knee osteoarthritis/ koa

## Abstract

Knee osteoarthritis affects millions of people worldwide. There remains a role for novel therapies to manage pain for patients who are unable or unwilling to undergo knee arthroplasty. A peripheral nerve stimulator (PNS) may be beneficial in this population. We present a case report of three patients who received temporary femoral or saphenous PNS and were either unwilling or unable to undergo knee arthroplasty. Two of the three patients reported significantly reduced pain and improved functioning. Our case report demonstrates that temporary PNS may offer a safe and effective treatment for chronic knee pain secondary to knee osteoarthritis.

## Introduction

Knee osteoarthritis affects more than 650 million individuals worldwide and is a leading cause of disability [1]. After an initial course of physical therapy and other conservative measures, current treatment for osteoarthritis-based chronic knee pain is mainly pharmacologic. There are limited interventional options with knee arthroplasty as a terminal choice [2,3]. However, not all patients are candidates for total knee arthroplasty, and some patients may not be interested in surgery. 

One alternative is the use of a peripheral nerve stimulator (PNS). This treatment is increasingly used in clinical practice to treat conditions such as peripheral neuropathy, complex regional pain syndrome, and other acute and chronic pain conditions [4,5]. There continues to be an active investigation into the mechanisms of action of PNS. The current common hypothesis is the gate control theory, first described by Melzack and Wall in 1965 [6]. This theory has been supported by PNS research showing stimulation of non-nociceptive Aβ fibers results in suppression of nociceptive processing [7]. The use of PNS is an effective option for pain management other than knee osteoarthritis. However, there is only one published case report detailing the use of a temporary PNS in a patient with knee osteoarthritis and chronic pain who refused total knee arthroplasty [8].

Therefore, we would like to present a case report of three patients undergoing temporary PNS for the treatment of osteoarthritic knee pain. This manuscript adheres to the CARE guidelines.

## Case presentation

Implantation procedure

**Figure 1:**
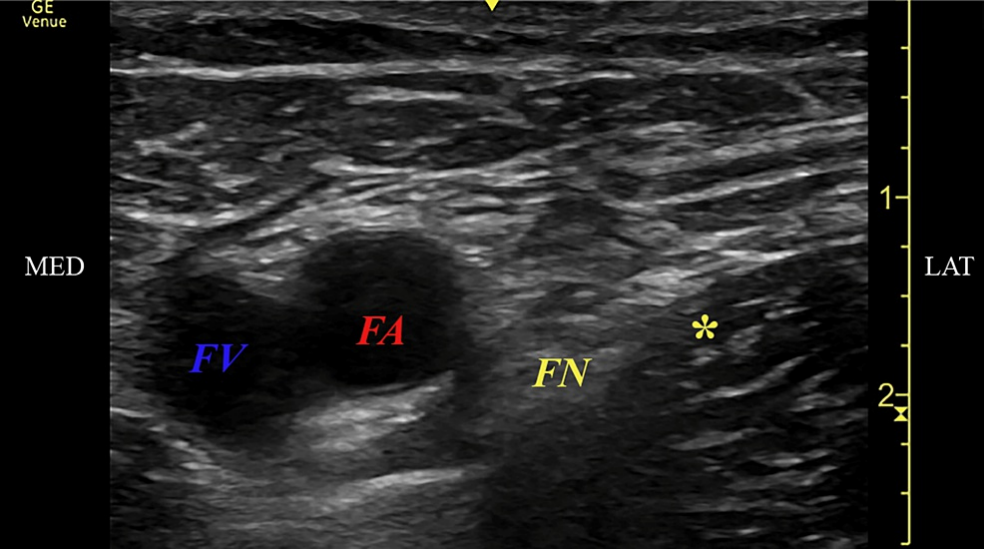
Ultrasound image of the femoral triangle using a linear array transducer Ultrasound of the femoral nerve (FN) at the inguinal crease. * Denotes the final location of the peripheral nerve stimulator (PNS). FA= femoral artery, FV= femoral vein, MED= medial, LAT= lateral

A femoral nerve PNS was performed with the patient in a supine position. The anterior thigh and groin areas were prepped with chlorhexidine and draped in a sterile fashion. The site of needle insertion was chosen just below the femoral crease, approximately 1-2 inches below the inguinal ligament. The skin and subcutaneous tissue over the groin were anesthetized by the infiltration of 1% lidocaine. A 12.5 cm, 20 g SPR Therapeutic Inc. introducer (SPRINT, SPR Therapeutics, Inc., Cleveland, OH, USA) needle preloaded with stimulating lead was inserted under ultrasound guidance with a linear array probe. Using an in-plane approach, the needle was directed towards the femoral nerve from the lateral to the medial side [9]. Once the tip of the needle was confirmed to be around 0.5-2cm from the femoral nerve, the lead was then attached to an external pulse generator. Stimulation was then delivered at approximately 100 Hz, amplitude (range: 0.2-20 mA), and pulse duration (range: 15-200 μsec). Care was taken to avoid motor stimulation, and the needle position was adjusted until the patient only reported sensory stimulation in the knee or lower thigh. The lead at that point was then deployed, and the introducing needle was withdrawn. The ideal ultrasound image and lead location are illustrated in Figure 1. Wound closure glue was applied at the exit site. The excess lead was trimmed, and the lead was attached to a connector block and again to the pulse generator.

For the saphenous nerve PNS, the patient was again placed in a supine position, and the skin over the area was prepped in a sterile fashion. A linear array ultrasound probe was placed transversely at the junction between the middle and distal thirds of the thigh. Using a lateral to medial in-plane approach, the percutaneous PNS lead was implanted 0.5cm from the saphenous nerve to enable selective activation of large-diameter sensory fibers. Patients tolerated the procedure well, and there were no apparent immediate complications or adverse effects seen in all three patients. 

Case 1

The patient was a 47-year-old female with a history of five years of chronic left knee pain secondary to knee osteoarthritis. Her pain was refractory to conservative therapy, including physical therapy, medication management with ibuprofen, diclofenac gel, and cannabidiol oil, as well as intra-articular injection therapy with steroids and hyaluronic acid. The patient reported a pain score of 7/10 on a numerical rating scale and noted that her pain was sharp and burning, non-radiating, and located in the anterior and medial parts of her left knee. Orthopedic surgery did not recommend surgical intervention, given her age. She was offered a temporary 60-day femoral PNS, which she agreed to pursue. The patient reported an improvement in her pain score to 2/10 immediately after the procedure. Two months after the procedure, her pain remained at a 4/10. She reported a substantial decrease in her ibuprofen usage (20 tablets to 2 tablets per week) and an increase in her ability to rock climb and hike as compared to prior. 

Case 2

The patient was a 47-year-old female with a history of four years of bilateral chronic knee pain secondary to knee osteoarthritis. Her pain was refractory to physical therapy and medication management. The patient initially had relative success with intra-articular steroid injections. However, she noticed that they were working for decreasing amounts of time. An intra-articular hyaluronic acid injection was trialed with no benefit. The patient reported a pain score of 7/10 and noted that her pain was dull, non-radiating, and located in the anterior, medial, and lateral aspects of her knees. Orthopedic surgery did not recommend surgical intervention, given her age and BMI of 45. The patient was offered a temporary 60-day left femoral PNS and agreed to proceed with this procedure. Immediately after the procedure, the patient reported her pain as a 0/10. Two months after the procedure, the patient reported continued 0/10 pain at rest and 2-3/10 with activity. She was able to stop all medication usage and had significantly improved activities of daily living, including driving and walking. 

Case 3

The patient was a 63-year-old male with a history of five years of left chronic knee pain secondary to knee osteoarthritis. The patient’s knee pain was refractory to physical therapy and medication management. The patient had previously had some success with intra-articular steroid injections for three years; however, they subsequently lost effectiveness. The patient also did not benefit from intra-articular hyaluronic acid injections. The patient reported that his pain score was a 4/10 at rest and a 6/10 with activity and noted that the pain was dull, non-radiating, and located over the entirety of the knee. The patient remained on oxycodone 5mg four times a day without significant improvement in his pain. The patient was uninterested in surgery and was offered a temporary 60-day femoral PNS. The patient agreed to proceed with this procedure. During the procedure, the patient experienced significant muscle contractions with stimulation. Due to this, the saphenous nerve was targeted until the patient experienced comfortable paresthesia without muscle contractions. Unfortunately, two weeks after the procedure, the patient reported uncomfortable cramping and inadequate coverage over his painful areas. The PNS was then removed without any complications.

## Discussion

This retrospective case series describes three patients with chronic knee pain secondary to knee osteoarthritis. As noted above, two out of three patients were treated with temporary femoral nerve PNS therapy delivered through a single percutaneous lead. One patient was treated with temporary saphenous nerve PNS due to muscle contractions with femoral nerve stimulation. Two out of three patients had improvement in functional status and pain score. One patient had no improvements in functional status or pain scores.

In our literature search, we found no reports of the use of femoral PNS for patients with knee osteoarthritis who were unable or unwilling to pursue knee arthroplasty. There has been one case report published detailing a patient with chronic knee pain secondary to knee osteoarthritis who refused total knee arthroplasty [8]. This patient received temporary PNS at both the saphenous and superior lateral genicular nerves. He reported 100% pain relief and improved functional status. Unfortunately, the patient was unable to receive the full duration of the 60-day PNS system secondary to lead dislodgement.

Femoral PNS is an effective option for pain management for other indications. Ruak et al. discuss the use of a temporary femoral PNS for a patient with post-amputation pain [10]. The patient reported a 60% improvement in residual limb pain, with significant improvements in his physical functioning, quality of life, and interference of his pain with daily life.

There have also been several studies showing the effectiveness of femoral PNS alone or femoral and sciatic PNS for acute postoperative analgesia after either total knee arthroplasty or anterior cruciate ligament reconstruction [11-14]. These studies showed a positive impact on pain scores and a reduction in opioid requirements with the use of PNS. While the mechanisms behind peripheral nerve stimulation are relatively unknown, research has shown that both spinal and supraspinal mechanisms are involved in pain relief with the use of PNS [15]. In our case, the use of PNS may have aided in the downregulation of both inflammatory and neuropathic pain.

There are limitations to our case study. First, this is a retrospective study, and some benefits may be attributed to the placebo effect. In addition, we chose to target only the femoral nerve, though anatomic reviews show that both the femoral and sciatic nerves carry the primary sensory innervation to the knee [16,17]. While all three of our patients had pain that was primarily limited to the anterior and medial aspects of the knee, there could be a role for femoral and sciatic PNS placement. Limitations and complications from temporary PNS are rare and include bleeding, infection, inability to achieve appropriate sensory stimulation and lead fracture or dislodgement, among others.

## Conclusions

Our cases demonstrate that femoral PNS is a viable option to manage chronic pain secondary to knee osteoarthritis in those who are unwilling to undergo knee arthroplasty or are poor candidates for surgery. Our results indicate that further clinical studies, such as a randomized controlled trial, are warranted.
